# Neuro-ophthalmologic findings of hypovitaminosis a in beef cattle: a retrospective study

**DOI:** 10.1080/01652176.2025.2546825

**Published:** 2025-08-13

**Authors:** Giulia Cagnotti, Cristina Giordano, Giorgia Di Muro, Sara Ferrini, Chiara Giudice, Giuliano Borriello, Antonio D’Angelo

**Affiliations:** a Department of Veterinary Sciences, University of Turin, Turin, Italy; b Department of Veterinary Medicine and Animal Sciences, University of Milan, Milan, Italy

**Keywords:** Bovine, blindness, seizures, vitamin A, neurology

## Abstract

Vitamin A in cattle is essential due to its antioxidant properties and importance in vision, immune function, reproduction, and cellular differentiation. This study investigates the clinical presentation, diagnosis, and treatment outcomes of hypovitaminosis A in 15 Piedmontese calves, referred to the Veterinary Teaching Hospital in Turin for vision impairment between 2016 and 2024. Neurological and ophthalmological evaluations revealed hypovision or blindness in 87% of cases, with additional epileptic seizures in 13%. Ophthalmological findings included papilledema, optic nerve atrophy, and retinal abnormalities, which were consistent predictors of poor prognosis for vision recovery. Serum vitamin A levels were below the reference range in all cases, confirming a primary dietary deficiency linked to the use of dry, preserved forages.Parenteral administration of vitamin A and dietary supplementation improved clinical outcomes in most cases, with 67% of affected calves achieving complete recovery. Histopathological analysis of one subject revealed ischemic degeneration presumably due to narrowed optic foramina caused by vitamin A deficiency. The findings emphasize the importance of early diagnosis through ocular and neurological assessments to prevent irreversible damage and economic loss in cattle herds. Prompt supplementation can mitigate health and productivity losses, emphasizing its critical role in herd management practices.

## Introduction

A crucial nutrient for cattle, vitamin A is essential because of its antioxidant properties and for maintaining normal vision, immune function, reproduction, and cellular differentiation. Hypovitaminosis A leads to health problems and diminished productivity in cattle. Vitamin A-deficient animals exhibit a variety of clinical signs: keratinization of epithelial tissues, immune dysfunction, increased susceptibility to infection, reduced reproduction, and slow growth rate. In young calves, which rely on the maternal transfer of vitamin A during both gestation and lactation, deficiency can cause perinatal deaths, weaken immune response and increase mortality, while in breeding animals, it can reduce reproduction and induce abortion (Frye et al. 1991; He et al. 2012; Sosa et al. 2024).

Vitamin A deficiency is a global health concern in cattle herds, especially in young growing animals on dry pasture or fed diets deficient in the vitamin or its precursors. Nonetheless, hypovitaminosis A remains underdiagnosed and undertreated in many cattle populations, primarily due to difficulty in diagnosis and in identification of early neuro-ophthalmologic signs (Constable et al. 2017). To our best knowledge, no studies to date have investigated the clinical neurologic and ophthalmologic presentation of hypovitaminosis A in cattle in Europe. With this case series we describe the clinical presentation, diagnosis, and treatment outcome of calves with hypovitaminosis A.

## Materials and methods

The medical record database of the Veterinary Teaching Hospital, Department of Veterinary Sciences, University of Turin was searched for cattle referred for vision impairment and a diagnosis of vitamin A deficiency between January 2016 and January 2024. Data were retrieved for signalment, neurologic and neuro-ophthalmologic examination, clinicopathological findings, including cerebrospinal fluid (CSF) analysis when available, treatment, and outcome. Patients were included if vitamin A serum concentration measurements were available and found to be below the laboratory reference range.

Clinical neurologic examination was performed by a board-certified neurologist (ADA) or a neurology resident in training (GC). Ophthalmologic examination was performed by an expert veterinary ophthalmologist (CG). The exam entailed dazzle and pupillary light reflex evaluation, slit lamp biomicroscopy, applanation tonometry, and indirect ophthalmoscopy. Each fundus was imaged with a retinal camera and the fundoscopic appearance was described.

Blood samples were obtained from the jugular or the coccygeal vein and placed into two tubes containing EDTA and a coagulation activator for complete blood count and biochemistry profile analysis. CSF was collected in empty tubes from the lumbosacral region with the animal in either sternal recumbency or standing position, as described by Mayhew (Mayhew and MacKay 2022).

The eyeballs and the optic nerves of one animal that did not recover were retrieved for histopathological examination at the time of slaughtering. Tissues were fixed in 10% neutral-buffered formalin and processed routinely for histopathology. The eye globe was vertically sectioned through the optic nerve, and both halves were embedded in paraffin for routine histological processing. Microtomic Sections 4-5 micrometers thick were cut from the paraffin blocks and stained with hematoxylin and eosin (Eosin G/Y alcoholic 0.5%, Diapath; Mayer’s Hemalum solution: Merck), and Schiff’s periodic acid for histological examination.

Descriptive statistics were determined for age and laboratory test results.

## Results

The study population was 15 Piedmontese cattle (12/15, 80% male; 3/15, 20% female) from four different herds. The median age was 5 months (interquartile range [IQR] 2.2–8 months).

The primary reason for presentation was consistent across all subjects: 13/15 (87%) presented with hypovision or blindness and 2/15 (13%) presented with hypovision or blindness associated with epileptic seizures. Many cases (5/15, 33%) were referred in August, 4/15 (27%) in October, 3/15 (20%) in February, and 2/15 (13%) in June.

Clinical evaluation was unremarkable. Neurologic examination in the field was generally similar: a disoriented mental state, a stilted gait with mild proprioceptive ataxia in all limbs. Additionally, pseudo-hypermetria in the forelimbs was observed in 3/15 cases. Due to the patients’ size and temperament, proprioception was assessed in 7/15 and found to be mildly reduced in all limbs. The menace response and direct and indirect pupillary light reflexes were bilaterally reduced or absent in 10/15 and 5/15 animals, respectively.

**Figure 1.**
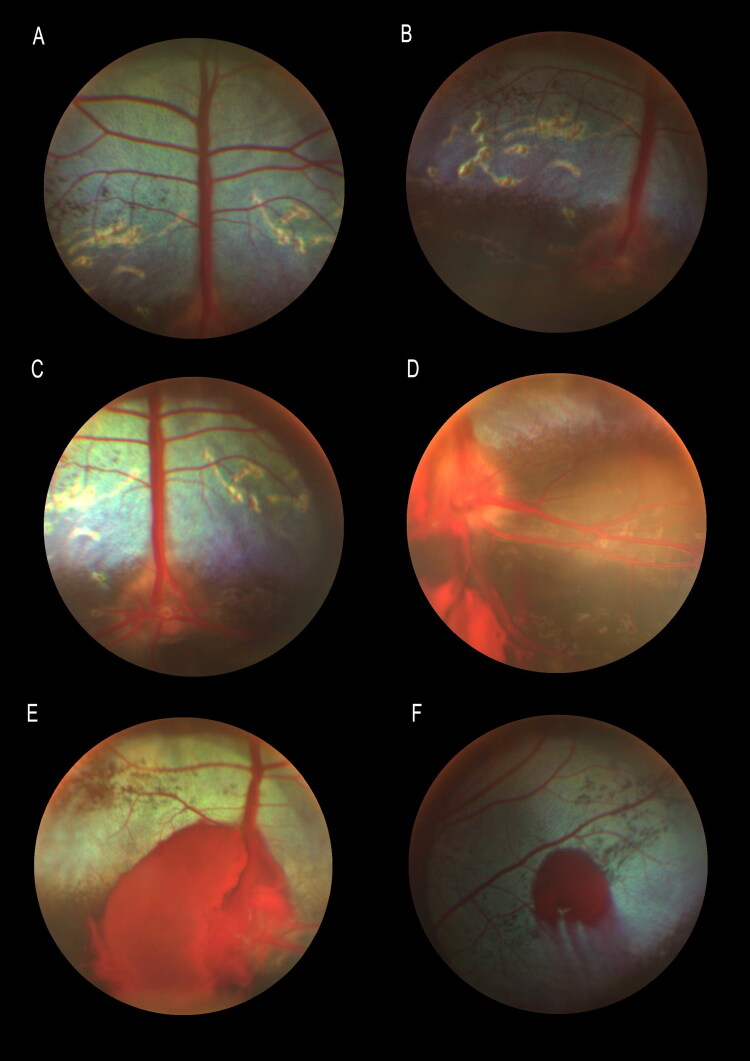
A: Retinal pigment epithelium disruption; B-C: retinopathy and optic nerve atrophy after papilledema; D: Papilledema, optic disc with indistinct and raised borders and large papillary and pre-retinal haemorrages; E-F: retinal hemorrhage.

Ophthalmologic examination of 7/15 animals revealed blindness in 5/7 and dramatically reduced vision in 2/7. Mydriasis was present in all subjects. No abnormalities of the anterior segment or intraocular pressure were noted; however, fundic examination showed varying degrees of papilledema or optic nerve atrophy, retinal blood vessel engorgement, papillary and peripapillary hemorrhages, and pigment disruption in the non-tapetal region (Figure 1).

A complete blood count and biochemistry panel revealed no gross abnormalities in 12/15 subjects, while 3/15 exhibited elevated creatine kinase (CK) activity (18780, 2280, and 882 U/L; reference interval 105–409 U/L) and increased aspartate aminotransferase (AST) activity (580, 221, and 134 U/L; reference interval 43–127 U/L).

Cerebrospinal fluid was collected in 10/15 animals. One sample was deemed inadequate for analysis due to blood contamination, while the remaining nine were within the normal limits. The median total nucleated cell count was 2 cells/mm³ (IQR 1–7; reference range <5 cells/mm³), the red blood cell count was 10 cells/mm³ (IQR 5–200), and the microprotein concentration was 20 mg/dL (IQR 14–43; reference range: <45 mg/dL).

Serum vitamin A was low on all animals (median 49 mcg/l, IQR 40-85,5; reference range 130-380 mcg/l). The affected calves received parenteral administration of vitamin complex containing vitamin A by intramuscular injection (dosage 440 IU/kg, Adisole A D E, Ceva Salute Animale s.*p.a.*). Dietary vitamin A supplementation was also provided for the herd. No other treatment or supplements were administered to the affected subjects or the herd. An improvement in clinical signs was noted in 10/15 cattle, with resolution of epileptic seizures and amelioration to complete recovery of vision. The subjects that presented with optic nerve atrophy on ophthalmologic evaluation did not recover vision.

To date, no other clinical cases within the herds have been identified since dietary vitamin supplementation.

**Figure 2.**
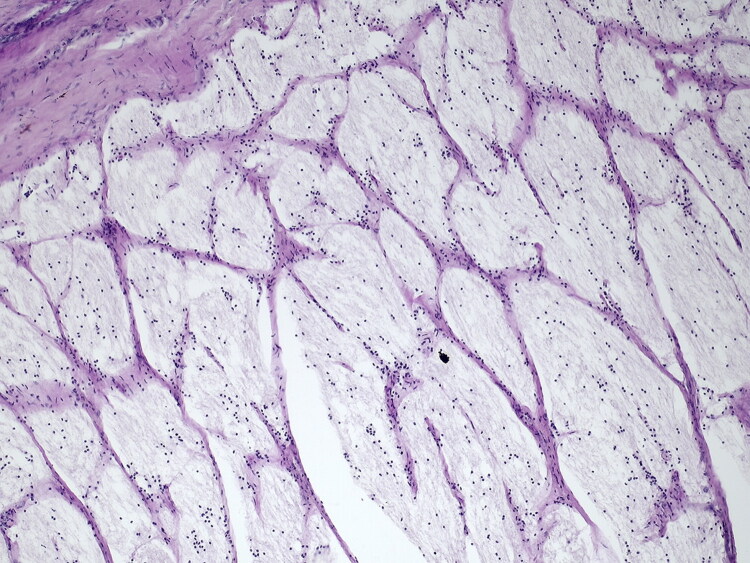
Periodic acid of Schiff staining, 20x. Detail of the optic nerve illustatring diffuse rarefaction with loss of nerve fibers and mild, diffuse gliosis.

Finally, histopathological examination of the eye globes was unremarkable, with no signs of retinal degeneration. However, the optic nerves displayed severe diffuse rarefaction with loss of nerve fibers and mild gliosis, consistent with secondary optic nerve degeneration due to stenosis of the optic foramen (Figure 2).

## Discussion

Cattle obtain vitamin A through the intestinal conversion of carotenoids present in green forage into retinol, the active form of the vitamin (Mackay et al. 2020). Hypovitaminosis A may be considered a primary disease, when cattle fail to receive adequate levels of this fat-soluble vitamin through the diet, or a secondary disease when the dietary supply is adequate, but intestinal absorption or metabolism is impaired (Constable et al. 2017).

Since no clinical or laboratory signs supporting a diagnosis of secondary deficiency of vitamin A (i.e. ill thrift and poor growth or diarrhea) were reported for the study population, a primary dietary deficiency may be assumed.

Our study population included calves with a broad age range, from 1.5 to 11 months. This variability is relevant when considering the pathogenesis of vitamin A deficiency. In younger calves, clinical signs are likely associated with maternal nutritional deficiencies during gestation or insufficient vitamin A intake from maternal milk. Conversely, in older calves, a proper dietary deficiency becomes the more plausible cause.

Primary hypovitaminosis A is prevalent in herds predominantly fed on dry, stored forages, which have low vitamin A levels due to degradation over time (Mackay et al. 2020). The incidence of clinical cases is consequently higher during the spring and winter months (Hill et al. 2009; He et al. 2012; Parker et al. 2017). We observed no clear seasonal pattern, as the cases were presented fairly regularly throughout the year. Hypovitaminosis A is often reported for Australia, where the climate differs markedly from that of Europe and northern Italy. This may explain the different seasonal pattern for our population.

Moreover, clinical signs of hypovitaminosis A usually manifest when serum/plasma vitamin A levels fall to <200 mcg/L (Mayhew and MacKay 2022). Livestock are known to be resistant to short-term deprivation because the liver stores vitamin A. Clinical signs may manifest from 6 months to 2 years of deficiency, resulting in considerable variability in the timing of presentation (Mackay et al. 2020). This may also explain the apparent discrepancy between our findings and the seasonality reported in the literature. In our study, hepatic vitamin A concentrations were not assessed, as all animals included, apart from their visual deficits, were otherwise clinically healthy, were supplemented with vitamin A supplemented and slaughtered several months later, which would have rendered any post-mortem hepatic evaluation irrelevant.

Growing beef calves are overrepresented in the literature, likely due to their higher growth rates and vitamin requirements compared to other breeds (Booth et al. 1987; Hill et al. 2009; He et al. 2012; Parker et al. 2017). We observed a similar predisposition in our study population, which included only Piedmontese breed calves. Several factors may influence the rate of carotenoid conversion into vitamin A in cattle, including potential breed-related differences, which may further explain the overrepresentation of the Piedmontese breed in this study (Frye et al. 1991). Another possible explanation for the predisposition of Piedmontese breed to hypovitaminosis A may lie in the stringent production regulations that prohibit the use of concentrated feeds in their diet from weaning to slaughter. The cattle are fed on dry and/or preserved forages, with an additional permitted use of simple feeds or mixtures of feeds that may be supplemented only with mineral-vitamin additives and other supplements. Therefore, the nutritional composition of the ration varies widely depending on climatic conditions.

Visual deficits were the most frequent reason for presentation. Peripheral blindness and papilledema are the first outward sign noted among young, growing animals with vitamin A deficiency. If addressed early in the course of deficiency, these conditions can be reversed with appropriate vitamin supplementation (Martins 2013; Cantile and Youssef 2016). The same conclusion can be drawn from our study population. Not all animals underwent fundus examination; however, those that did displayed similar conditions, with variable degrees of papilledema, recovered vision after appropriate treatment. Differently, optic nerve atrophy was a consistent element predicting persistently impaired vision. Epileptic seizures have been associated with hypovitaminosis A in calves; clinical signs have resolved with vitamin A supplementation (Mayhew and MacKay 2022), as we also observed.

The only bloodwork abnormalities we observed were elevated CK and AST levels in three animals. All were experiencing epileptic seizures at the time of evaluation, which explains the levels, as documented in the literature for both small and large animals (D’Angelo et al. 2015; Paltrinieri et al. 2017). Indeed, the intense and sustained skeletal muscle contractions during epileptic seizures lead to mechanical stress and disruption of the sarcolemma, resulting in leakage of CK and other intracellular enzymes into the bloodstream (Hoffmann and Solter 2008).

CSF analysis resulted normal in all animals diagnosed with hypovitaminosis A, which aligns with previous findings (Constable et al. 2017). Increased CSF pressure has been suggested as an indicator of vitamin A deficiency, with elevated pressure (>200 mm Hg) considered suggestive of hypovitaminosis A according to one study (Dbanapalan et al. 1992). Since CSF pressure was not measured, we are unable to corroborate the findings reported elsewhere. Nevertheless, this diagnostic technique is not routinely employed owing to its invasive nature, the need for animal restraint, and the wide variability in both healthy and diseased animals. Such variability can be due to fluctuation in venous pressure, posture, and hydration status, making it difficult to draw definitive conclusions based on the results obtained (Scott 2004).

Several pathogenetic mechanisms have been reported to cause the neuro-ophthalmologic manifestations of hypovitaminosis A in young cattle. In growing animals, vitamin A deficiency affects bone growth and remodeling, as well as CSF absorption. Vitamin A exerts a stimulatory effect on osteoclastic activity. Its deficiency leads to inadequate resorption of endosteal bone, which, at the level of the presphenoid bone, manifests as narrowing of the optic canal in the dorsoventral plane. Prolonged vitamin A deficiency results in progressive narrowing of the optic canal due to continued deposition of new bone on its dorsal surfaces and failure of bone resorption from the lateral and ventral aspects. This causes compression and subsequent ischemic necrosis, atrophy, and astrogliosis, particularly in the intraosseous portion of the optic nerve, as identified on histopathological evaluation of samples from one animal in this study. Wallerian degeneration of the optic chiasm and optic tracts may also occur. Over time, retrograde degenerative changes result in the loss of retinal ganglion cells and their axons, retinal atrophy, and gliosis (Summers et al. 1995; Cantile and Youssef 2016). The skull abnormalities associated with hypovitaminosis A could not be confirmed because complete anatomopathological examination was not performed.

In addition, vitamin A is essential for normal epithelial tissue differentiation (Frye et al. 1991). Hypovitaminosis A may result in collagenous thickening of the dura mater, leading to mechanical obstruction of CSF drainage at the arachnoid villi (Cantile and Youssef 2016). The obstruction increases CSF pressure, clinically manifesting as papilledema of the optic disc, as observed in several subjects in our study population.

Maternal deficiency of vitamin A during pregnancy has also been associated with multiple systemic anomalies in calves, including ocular and cardiac malformations (Millemann et al. 2007). No macroscopic ocular malformations were identified in the study population. Cardiac evaluation and anatomopathological examination of the heart were not performed. No clinical signs indicative of cardiac malformations, such as exercise intolerance, abnormal heart rhythm, or heart murmurs, were observed. While serum vitamin A concentration in the dams of the calves was not measured, the absence of clinically evident cardiac and ocular malformations does not support this pathogenetic mechanism for the clinical signs in the calves.

Hypovitaminosis A is implicated in defective rhodopsin synthesis. Rhodopsin is a light-sensitive receptor protein involved in visual phototransduction in rod cells under dim light conditions. This leads to night blindness, or nyctalopia (Martins 2013). The ultrastructural lesion involves swelling, followed by fragmentation, of the lamellar discs within the inner segments of the rod cells where rhodopsin is stored. This condition can be reversed within 2 weeks by vitamin A supplementation, provided the inner segments have not been irreparably damaged (Cantile and Youssef 2016). Appropriate treatment led to improvement or complete resolution of clinical signs in most animals, indicating that the prognosis of hypovitaminosis A depends on the severity and duration of the condition. Reports in the literature describe variable outcomes in terms of visual recovery, with some cases showing persistent deficits despite supplementation. Our findings underscore the importance of early diagnosis and timely intervention as key factors in ensuring a favorable prognosis. Prompt treatment appears to enhance the likelihood of visual recovery, thereby improving overall animal welfare and mitigating economic losses associated with delayed recognition of the condition.

In conclusion, although hypovitaminosis A can have a profound impact on cattle health and productivity, it remains underdiagnosed and undertreated in many cattle populations, primarily due to difficulty in diagnosis and absence of clear clinical symptoms during its early stages. Ocular manifestations are major indicators of hypovitaminosis A in cattle and serve as early warning signs that can be detected in the field. Early detection and appropriate supplementation can prevent permanent lesions and mitigate economic loss in cattle herds.

## Supplementary Material

Supplemental Material

## Data Availability

The datasets generated and/or analyzed during the current study are available from the corresponding author upon reasonable request.
