# Supplementary figures and images for: Neuro-ophthalmologic findings of hypovitaminosis a in beef cattle: a retrospective study
Source: Vet Q. 2025 Aug 13;45(1):2546825. doi: 10.1080/01652176.2025.2546825 (PMC12351754; doi:10.1080/01652176.2025.2546825)

Supplementary Figure 1: Periodic acid of Schiff staining, 20x. Detail of a normal bovine optic nerve


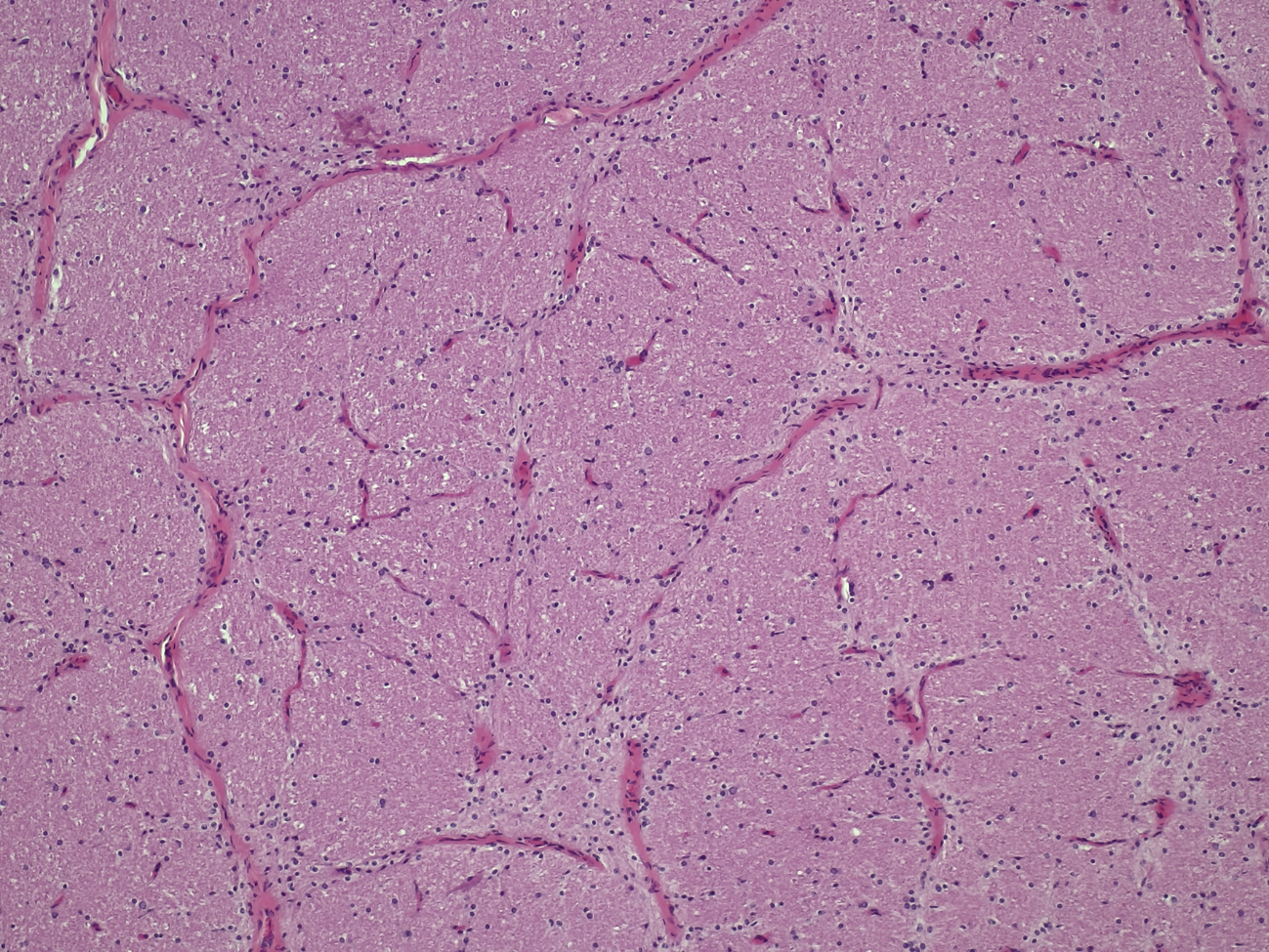

Supplement: Supplemental Material [file TVEQ_A_2546825_SM7376.docx]
